# The trade of glass beads in early medieval Illyricum: towards an Islamic monopoly

**DOI:** 10.1007/s12520-017-0583-5

**Published:** 2018-01-12

**Authors:** Elisabetta Neri, Bernard Gratuze, Nadine Schibille

**Affiliations:** IRAMAT-CEB, UMR5060, CNRS/Université d’Orléans, 3D, rue de la Férollerie, 45071 Orléans cedex 2, France

**Keywords:** Glass beads, Early Middle Ages, Illyricum, Recycled natron glass, Black lead glass, Islamic soda ash glass, Cobalt, Nickel, Zinc, Indium, Lead tin calx, Commercial network, Balkans

## Abstract

The trade of glass beads has long been assumed to have been under Islamic dominance during the early centuries following the Arab conquest of the Middle East, judged by the prevalence of Islamic beads in the archaeological contexts from Viking Scandinavia to medieval Morocco. This paper explores the impact of the Byzantine-Slavic transition on the use and by extension trade of glass beads in the Balkans from the seventh to the ninth century CE. A series of 48 glass beads and 4 vessel fragments from two excavated sites in modern day Albania have been analysed morphologically, technologically and chemically by LA-ICP-MS. The seventh-century beads from Lezha have typological parallels among central European assemblages and are made from recycled natron-type glass. The presence of a high lead-iron-natron variant is of particular interest as it potentially reflects a regional production. The ninth-century beads from Komani are made from soda-rich plant ash glass from the eastern Mediterranean and Mesopotamia and correspond to an Islamic typology. The chronological and geographical differences are reflected in the distinctive cobalt sources used for the two groups. While the beads from Lezha are coloured with a cobalt not correlated with any particular element, the cobalt source of the Komani samples is associated with zinc, typical of Islamic glass making. It thus appears that the supply of beads during the seventh century when the Balkans were under Slavic occupation relied on regional production and recycled material, and that a long-distance trade with the eastern Mediterranean was revived following the Byzantine re-conquest of the south-eastern Adriatic in the ninth century. Intriguingly, the Albanian finds confirm the Islamic control of the production and trade of glass beads during this period and highlight the mediatory role of the Byzantine Empire.

**Electronic supplementary material:**

The online version of this article (10.1007/s12520-017-0583-5) contains supplementary material, which is available to authorized users.

## Introduction

The multidisciplinary study of glass beads in early medieval contexts has proved particularly fruitful to elucidate commercial and cultural networks (Pion 2014; Pion and Gratuze 2016; Koleini et al. 2016; Wood 2012; Dussubieux et al. 2008; Dussubieux and Soedewo 2016). It has been shown that the chemical signature of glass beads is linked to their morphology and technology, which in turn are connected to geo-cultural traditions (Pion and Gratuze 2016). In early medieval Europe, for example, only wound beads, manufactured by rotating molten glass around a mandrel (metal rod), were produced. Workshops of wound beads are documented in Lombard Italy (e.g. Aiano Torraccia: Cavalieri and Giumlia-Mair 2009), in continental Europe during Merovingian times (e.g. Maastricht, Wijnaldum and Huy: Pion 2014), in Viking Scandinavia at Haithabu, Paviken and Ribe (Sode 2004; Callmer and Henderson 1991), in the Balkans at Preslav, and in the ninth- to tenth-century Caucasus (Bezborodov 1959). Drawn beads obtained from segmented tubes, on the other hand, are more common in Egypt, in the Levant and in India (Francis 1990, 2002, 2004). There are two main methods of drawn bead production: hot and cold cutting. Workshops of hot-cut beads have been found in Egypt, at Kom-el-Dikka near Alexandria, dating to the fifth and sixth centuries, and at seventh- to eleventh-century Fustat (Arveiller-Dulong and Nenna 2011). No archaeological evidence of workshops for cold-cut beads have as yet been identified in the eastern Mediterranean. Anthropological studies attribute this technique to the Indo-pacific tradition of the ‘lada method’ still practised today (Francis 1990, Fig. 2 and pl. III, b; Kanungo 2004). According to this manufacturing technique, beads are produced by drawing the glass using a tapered iron tube with a large end, the so-called lada, into glass cylinders that are then cold-cut into beads (Francis 2002; Pion and Gratuze 2016).

The study of Merovingian and Lombard beads showed that most of the beads produced in central Europe between the sixth and eighth century were wound beads made from natron type glass derived from the primary sources in Egypt and the Levant or from recycled cullet (Mathis et al. 2013; Pion and Gratuze 2016; Pion 2014; Poulain et al. 2013; Verità 2012). In addition to these locally produced wound beads, segmented drawn beads produced from soda plant ash glass and small Indo-Pacific beads of high aluminous soda glass were simultaneously imported (Pion and Gratuze 2016). This provides ample evidence of long-distance trade networks that connected western Europe with the eastern Mediterranean and the Indian Ocean during the fifth and early sixth century CE. These eastern imports ceased sometime during the later sixth century, the reasons for which are still unknown (Pion and Gratuze 2016; Calligaro and Périn 2013). Following an interval of about two centuries, the long-distance trade of glass beads experienced a revival in the ninth century, after the Abbasid capital and political focus had shifted eastwards from Damascus to Baghdad (Sode 2004; Robertshaw et al. 2010). This reorientation arguably played a role in the consolidation of networks of exchange and the influx of Mesopotamian beads to the Mediterranean region and as far as Viking Scandinavia (Callmer 1977; Steppuhn 1997; Steppuhn 1998; Sode 2004).

This paper presents the first extensive study of medieval glass beads from south-western Illyricum in modern-day Albania, combining typological with analytical methods. This geographical region is important because it occupies a central position and was a crucial commercial hub that connected the eastern Mediterranean with western Europe. The glass beads analysed are from two well-dated necropoleis in the Drin valley. The material from Lezha is attributed to the seventh century, while the beads from Komani come from ninth- to tenth-century contexts. This is a critical period in the history of the Balkans as it encompasses the Slavic invasion in the seventh century and the Byzantine re-conquest in the ninth (Bavant 2004; Cheynet 2006; Popovic 1980; Lemerle 1980; Fine 1983). The relationship between the vitreous materials and bead morphologies of the two sites can thus reveal how the transitional period impacted the trade of beads and the organisation of supply more generally (for previous studies on glass from the Balkans see, for example, Cholakova et al. 2016; Egorkov 2006; Jennings 2010; Schibille 2011; Šmit et al. 2012).

**Fig. 1:**
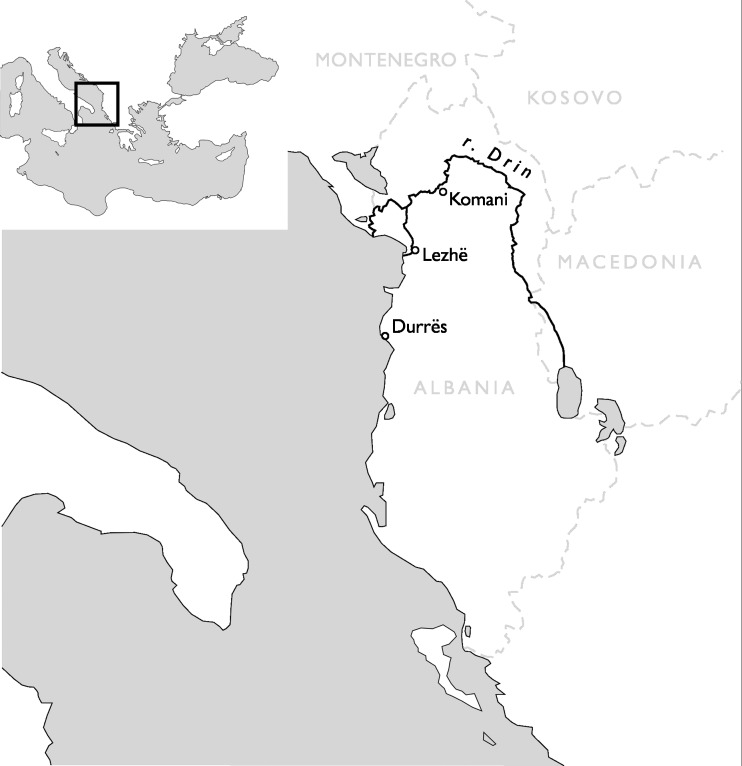
Map of Albania showing the location of Lezha (Lezhë) and Komani in the Drin Valley

**Fig. 2:**
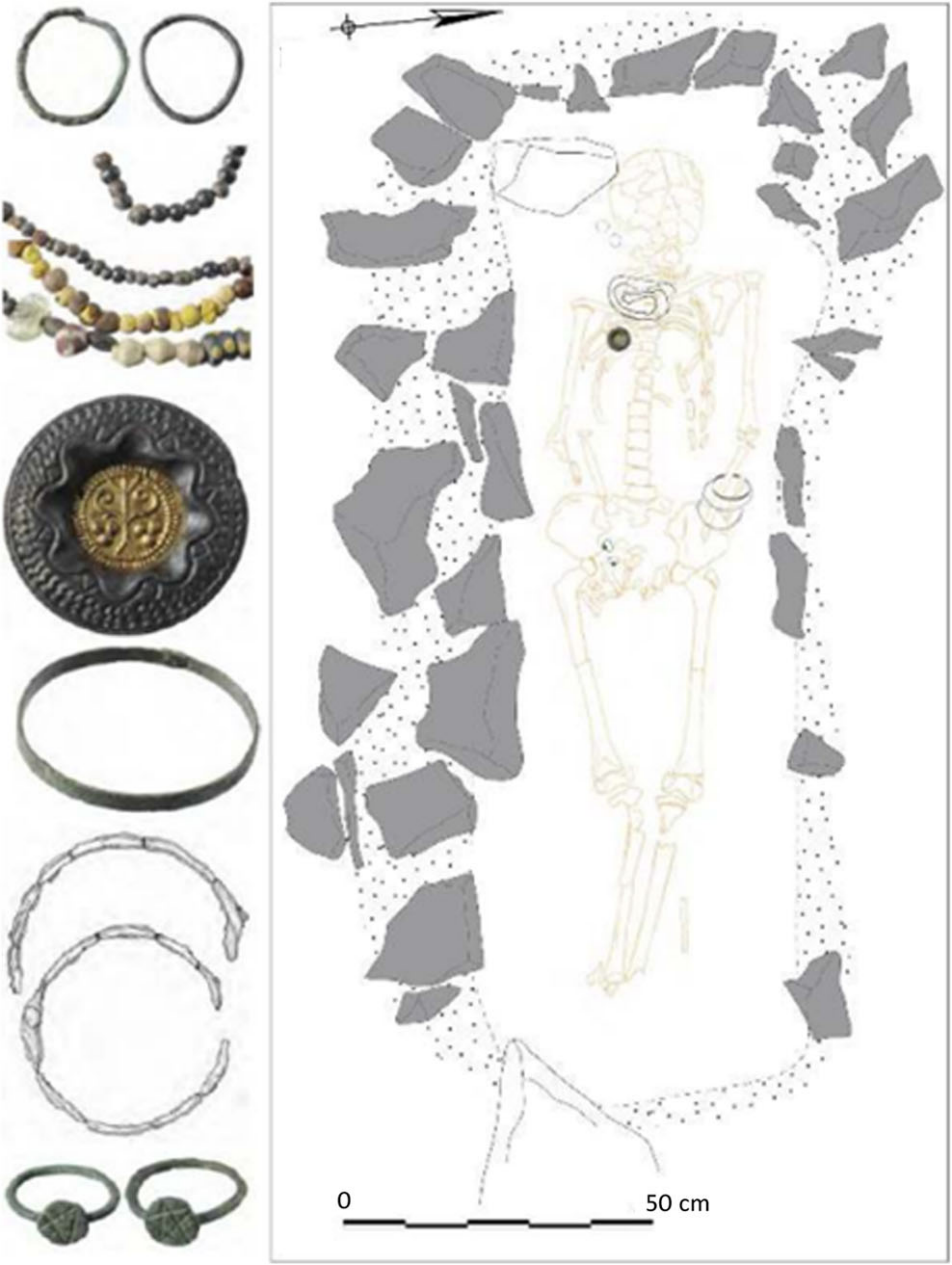
Seventh-century dressed inhumation (tomb T157) at Lezha (Nalbani et al. 2016, photo: Didier Dubois)

**Fig. 3:**
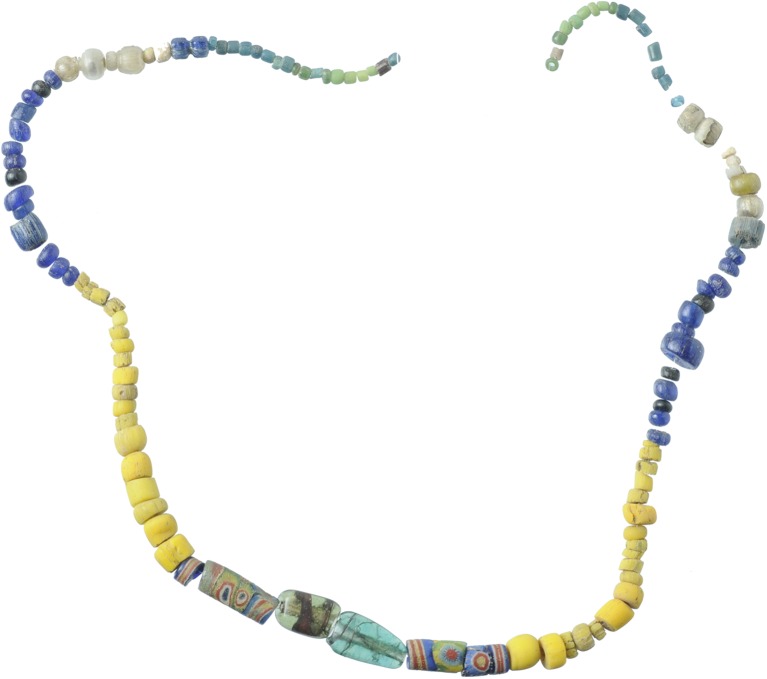
Necklace from a ninth- or tenth-century tomb (T319) in Komani (photo: Didier Dubois)

Forty-eight beads and four vessel fragments were selected from the necropoleis of two late antique foundations on the river Drin, the coastal town of Lezha and the town of Komani located further inland (Fig. 1). Lezha (Alessio, Lissos, 41.78 N, 19.64 E) was a long-lived (sixth to sixteen century CE) fortified settlement with a seventh-century church and an associated cemetery *extra muros* used from the sixth to the twelfth century. Komani (Koman, 42.08 N, 19.82 E), by contrast, was a much larger town with six churches and an extensive necropolis. There is evidence of two distinct periods of urban development, the first during the sixth and seventh century, and a second phase in the ninth and tenth century. The site was finally abandoned in the thirteen century (Nallbani et al. 2016a). The eighteen beads from Lezha were found in the context of dressed inhumations (Fig. 2) dated to the seventh century CE based on the associated finds, and two medieval samples from an Islamic grave (Table 1) (Nallbani et al. 2016a). The 33 beads from Komani were part of the grave goods from six different tombs, two of which have been dated by ^14^C to the ninth or tenth century. The other graves and grave goods can be attributed to the same period by association (Nallbani et al. 2016b). In three cases, entire necklaces were found intact (Fig. 3). In addition, four fragmentary liturgical glass vessels from the annex of the main church and from the settlement around the church of St. George were analysed for comparative reasons. While the beads were used to demonstrate status and prestige and were culturally associated with Slavic traditions (Wood 2012), the lamps and *unguentarium* from the ecclesiastical contexts are associated with the Byzantine religious élite (Cheynet 2006). The consideration of the vessel fragments within this study thus allows us to relate the objects types to specific socio-political contexts.

**Table 1:**
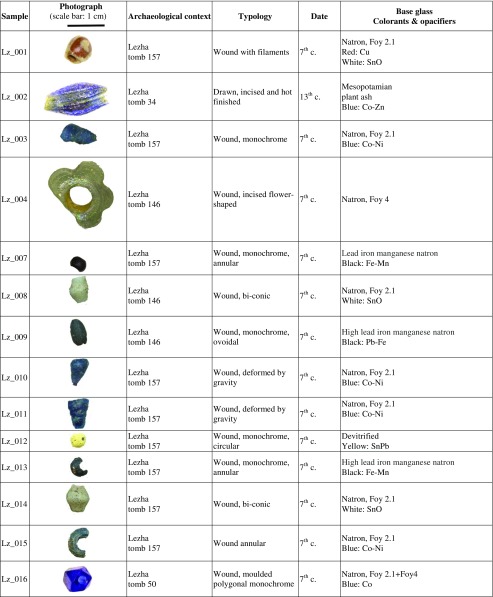

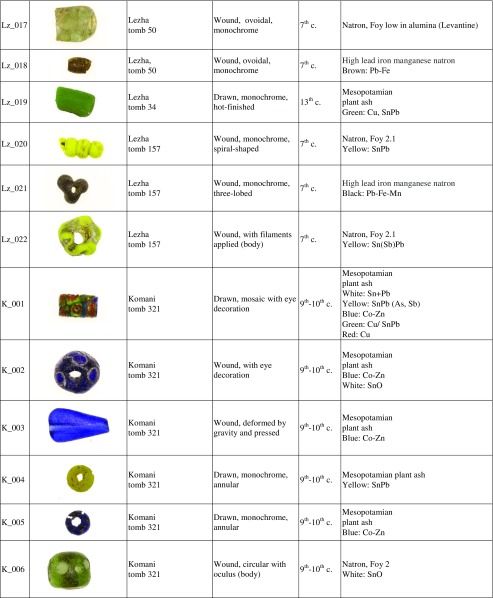

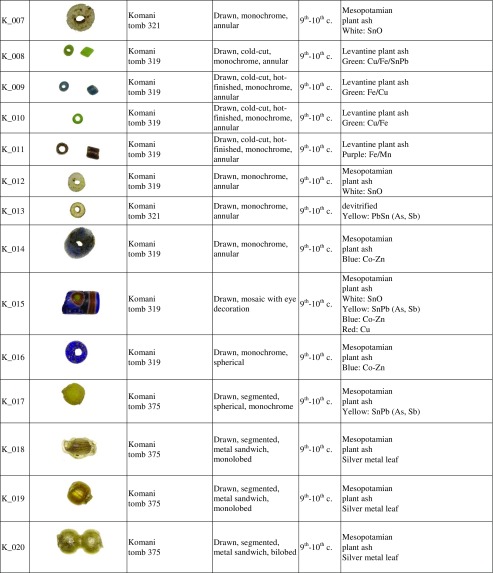

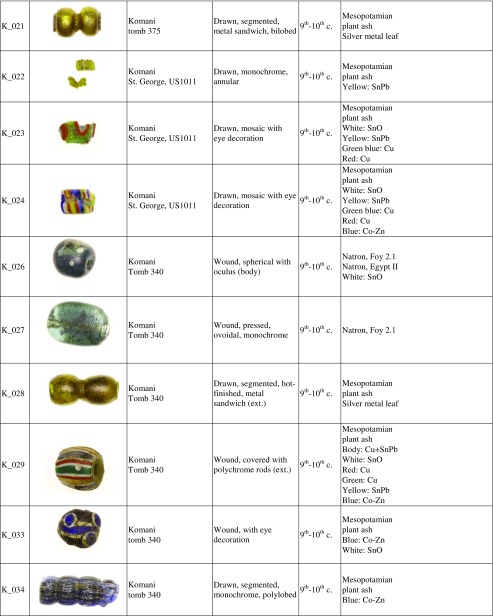
Glass beads from Lezha and Komani

## Material and methods

The samples analysed in this study were selected to cover the entire range of different typologies, colours and techniques identified among the assemblages from the two necropoleis at Lezha and Komani (Table 1). The cleaned but otherwise unprepared glass beads and fragments were analysed by Laser Ablation Inductively Coupled Plasma Mass Spectrometry (LA-ICP-MS). The analyses were conducted at the Centre Ernest-Babelon of the IRAMAT (Orléans), using a Thermofisher Element XR combined with a Resonetic UV laser microprobe (ArF 193 nm) for the direct analyses of solid samples (Gratuze 2016; Schibille et al. 2017). Single-point analyses were carried out with a laser beam diameter of 100 μm, operated at 5 mJ and a pulse frequency of 10 Hz. A pre-ablation time of 20 s, occasionally increased to 40 s to ensure the removal of possible corrosion layers or other surface contaminations, was followed by 30 s collection time. Blanks were run after every ten samples to determine the offset. The response coefficient (k) for each element was calculated based on a set of five glass standards (NIST SRM610, Corning B, C, D and APL1, an in-house standard) to convert the signals into quantitative data. Corning A and NIST 612 glass standards were systematically measured at regular intervals to determine accuracy and precision. The analytical precision reflected in the relative standard deviation (σ) was generally better than 5% for most elements and accuracy was mostly better than 10% (Supplementary material, Table S1).

## Results

### Typology and geographical distribution

The seventh-century beads from Lezha are all wound beads, but vary in shape and manufacturing technique, including monochrome beads of various shapes (spherical, annular, spiral-shaped, bi-conic), monochrome beads with applied filaments, pinched flower-shaped or three-lobed and moulded beads (Table 1). All these types are widely attested in Merovingian (Pion 2014), Lombard (Giostra 2012) and Alaman necropoleis (Koch 1977; Giesler-Müller 1992). This is in contrast to the assemblages from two Serbian sites of Viminacium and Singidunum where drawn beads are prevalent (Ivanišević et al. 2006).

The ninth- and tenth-century beads from Komani and the two medieval beads from Lezha vary likewise greatly in shape (umbilical, annular, tubular, mosaic, metal sandwich) and colour (yellow, blue, white), but they are mostly drawn and hot-cut, with or without a hot finish (Table 1). Similar drawn and hot-cut beads have been found in sixth- and seventh-century Serbian contexts (Ivanišević et al. 2006), among fifth- to sixth-century Merovingian assemblages (Pion 2014), a sixth to seventh-century southern Italian necropolis (Corrado 2012), some ninth- to eleventh-century Viking sites (Sode 2004) as well as across the Islamic Mediterranean (Francis 2004). A handful of small beads from a necklace retrieved from a tenth-century tomb are cold-cut and apparently produced with the ‘lada method’ (K_008, K_009, K_010, K_011). Indian cold-cut beads are known to have been imported to western Europe up to the sixth century (Pion and Gratuze 2016). The beads from Komani suggest that Indian ‘lada beads’ might have been imported to the Mediterranean region as late as the tenth century. Several types of wound beads from Komani such as the spherical beads with eye decorations (K_033) or polychrome rods (K_029) show similarities with the wound Islamic beads excavated at different Viking sites (Sode 2004; Callmer and Henderson 1991; Steppuhn 1997; Steppuhn 1998). The colourless ovoidal flat bead (K_027) and the colourless beads decorated with a white oculus (K_006, K_026) usually occupy the most prominent place within the necklace (Fig. 3).

**Fig. 4:**
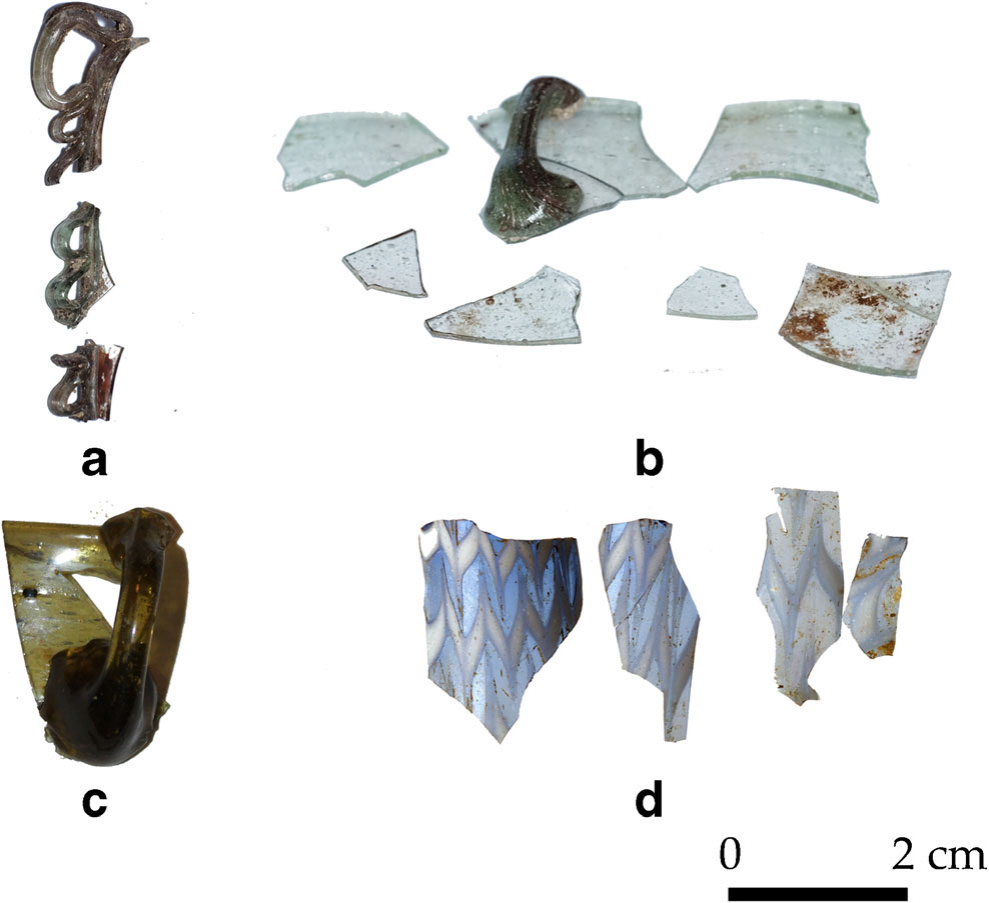
Glass artefacts from Komani. **a** Snake trailed lamp (similar to type 3.ii in Antonaras 2008). **b** Lamp with handle (similar to type 4 in Antonaras 2008). **c** Lamp with handle (similar to type II.i in Antonaras 2008). **d** Spindle-shaped unguentarium

The glass fragments included in this study can be attributed to ecclesiastical lamps, dated typologically to the fifth to eleventh century (Fig. 4a–c) (Antonaras 2008). While they are relatively common elsewhere, for instance in Thessaloniki, this is the first time they have been identified in Albania (Antonaras 2008, Jennings 2010). Finally, the last fragment with a marvered decoration belongs to a spindle-shaped *unguentarium*, usually dated to between the tenth and fourteenth century (Fig. 4d) and is linked to an Abbasid production. A similar object was found in Montenegro (Antonaras 2010a).

### Chemical signature of the beads

**Fig. 5:**
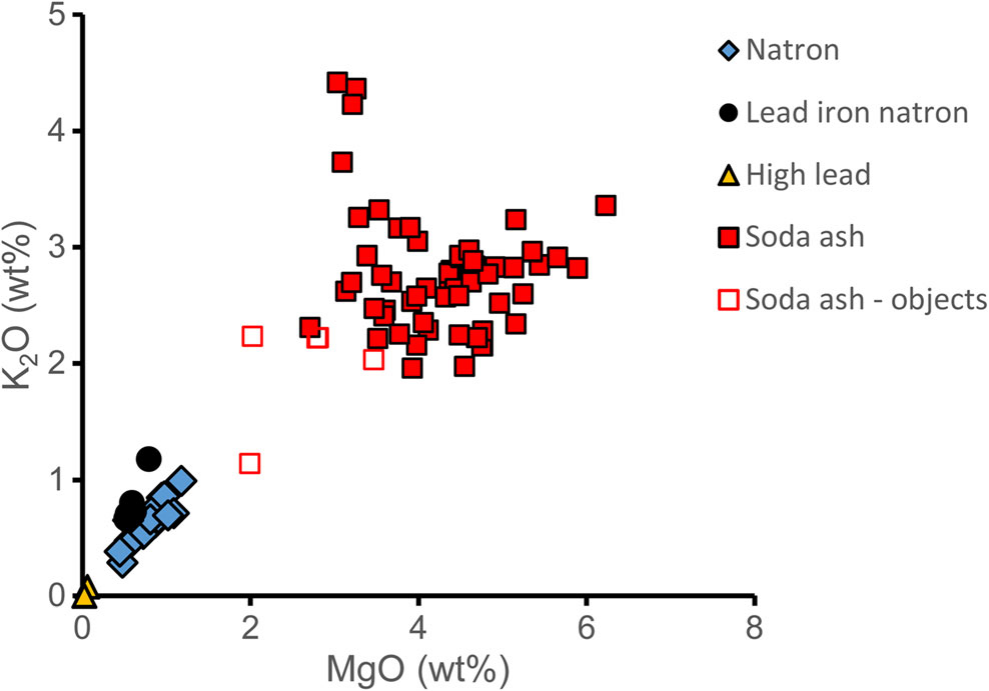
Potassium versus magnesium oxide concentrations, distinguishing the glasses according to the fluxing agents used

The LA-ICP-MS analysis of the beads (Table 2) clearly identifies two distinct groups according to the alkaline source that correspond to the chronology of the beads. Whereas all the seventh-century beads are natron-type glasses, the majority of the ninth- and tenth-century samples show a soda-rich plant ash signature, reflected in high potassium, magnesium (Fig. 5) and phosphorus oxide contents. Two opaque yellow beads (Lz_012, K_013,) have unusually high lead contents (PbO 70%) with very low quantities of formers and modifiers. Similar compositions have been observed in some Islamic green and yellow beads at Al-Basra (Robertshaw et al. 2010) and the Serçe Limani shipwreck (Brill 2009). In the case of the Albanian beads, the exceptionally high lead content could be the result of pure lead stannate, used as a half-product such as ‘anima’ known from Venetian recipes (Moretti and Hreglich 1984). A crucible with comparable residues of lead stannate for bead-making was retrieved from the Merovingian site of Schleitheim (Heck et al. 2003). However, it cannot be ruled out that the two beads represent highly devitrified lead stannate coloured glass, but the low phosphorus contents make this less likely.

**Table 2:**
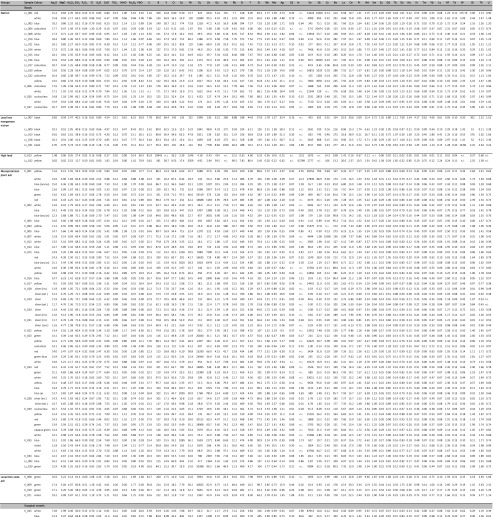
LA-ICP-MS data of the Lezha and Komani samples. Major and minor oxides [wt%], including chlorine, and trace elements [ppm]; *n.i.* not identified

#### Natron glass beads

The majority of the seventh-century wound glass beads, as well as three ninth- to tenth-century colourless beads (*n* = 12) have a natron-type base glass with elevated levels of magnesium, titanium and iron, associated with slightly increased vanadium, chromium and zirconium. These characteristics correspond to the so-called Foy-2 compositional group (Foy et al. 2003). Foy-2 glass appears to have been a relatively widespread glass type during the sixth and seventh century and is believed to be of an Egyptian provenance (Nenna 2014; Ceglia et al. 2015; Cholakova et al. 2016; Neri et al. 2017; Schibille et al. 2017).

**Fig. 6:**
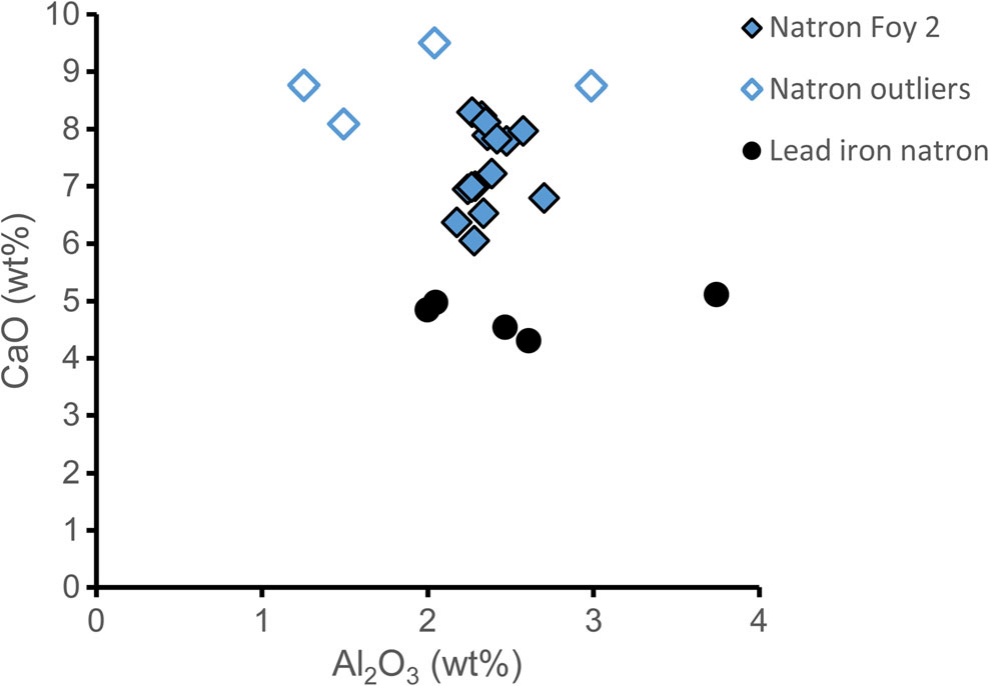
Calcium and aluminium oxide concentrations of the natron-type glasses, reflecting different silica sources

There are four outliers in terms of the elements associated with the silica sources (Fig. 6). The white glass of the spherical wound bead (K_026) has high calcium and low aluminium oxide contents, high chromium, magnesium, titanium and low strontium levels. This profile is typical of so-called Egypt II glass that was first identified among eighth- and ninth-century Islamic glass weights from Egypt (Gratuze and Barrandon 1990). One colourless wound bead (Lz_017) has a Levantine composition, except for its lower alumina content (1.25%). The flower-shaped wound bead (Lz_004) with equally low alumina (1.5%) and high antimony oxide (2.5%) concentrations bears similarities with Roman antimony decoloured glass, but for its relatively high lime concentration (Jackson 2005). The somewhat unusual trace element patterns, including low titanium but high zirconium, relatively low strontium and elevated copper levels suggest some degree of recycling and mixing of Roman antimony glass with another glass type. The polygonal transparent blue bead (Lz_016) has high antimony oxide (2%) levels, possibly as a result of recycled blue Roman mosaic tesserae opacified with antimony. Some of the compositional variations are probably due to the recycling and mixing of new base glass with tesserae and cullet of various origins. In particular, the traces of cobalt (CoO 0.01–0.03%) and copper (CuO 0.15–0.5%) in colourless or yellow beads and elevated phosphorus and potassium contents (e.g. K_026) can indicate the recycling of coloured cullet (Table 2).

Judging from the compositional analysis, the colourants and opacifying agents used include tin oxide to obtain the white beads and lead stannate to obtain yellow, both are typical of the early medieval period after the fourth century, when tin increasingly replaced antimony as opacifier (Tite et al. 2008). The blue beads are coloured with cobalt (500–900 ppm). The cobalt used for the natron-type beads is not unambiguously associated with any particular impurities, which is a trademark of Roman cobalt sources (Gratuze et al. 1992). One sample (K_026) has a low cobalt to nickel ratio (4:1) that is congruous with a cobalt source associated with elevated nickel that appears to have been exploited since the sixth century (Schibille et al. 2017).

The black glass beads from Lezha (*n* = 5) have a peculiar composition with high lead (PbO 28–34%) and iron (FeO 7.7–15.2%) contents, similar to some sixth- to seventh-century Merovingian beads (Poulain et al. 2013; Mathis et al. 2013; Pion and Gratuze 2016). They are probably the result of a mixture of recycled natron glass with a high lead and iron component, introduced in the form of lead and iron oxides or metallurgical scrap (Mecking 2013; Gratuze et al. 2003). The trilobed bead (Lz_021) appears to be somewhat of an outlier, differing not only in shape but also in composition from the other black beads. It has significantly lower soda contents, while manganese and aluminium are notably higher. These features suggest that this bead is the product of a different secondary workshop.

#### Soda ash glass

**Fig. 7:**
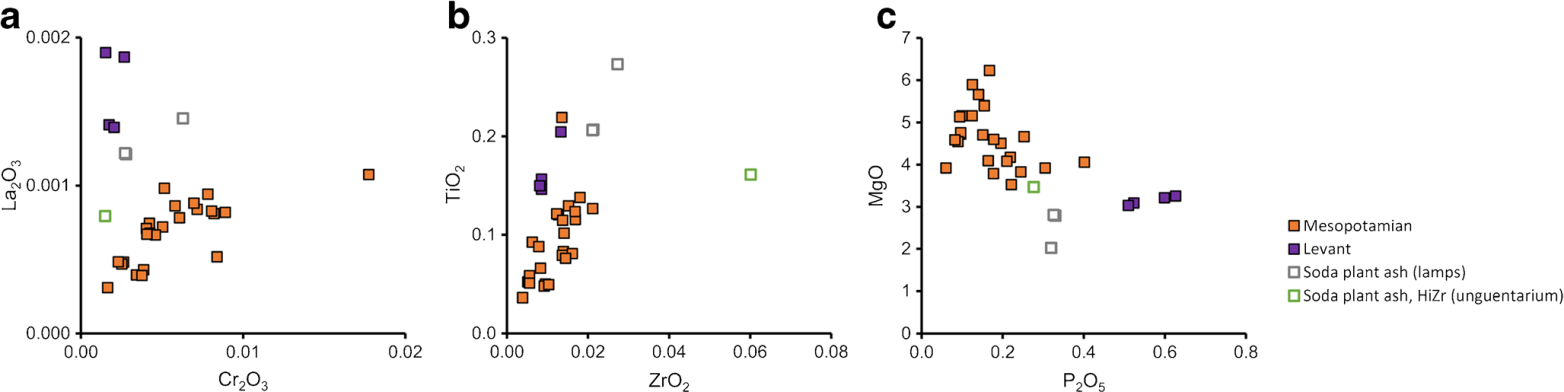
Chemical profile of the plant ash glass beads and objects from Lezha and Komani. **a** Lanthanum versus chromium concentration and **b** titanium versus zirconium concentration distinguish different silica sources; **c** phosphorus versus magnesium contents separate the alkali sources. One data point is shown for each bead, for the metal leaf beads an average was calculated, in the case of the multi-coloured mosaic beads the measurements of the blue glass segments are represented

The ninth- to tenth-century drawn and wound beads are made from soda plant ash glass, characterised by higher potash and magnesia contents (> 2%) associated with the plant ash component. Two sub-groups can be distinguished on account of their varying lanthanum, chromium, titanium and zirconium contents that reflect different silica sources (Fig. 7a, b). The different silica groups also exhibit differences in relation to the alkali contents with distinct magnesium and phosphorus ratios (Fig. 7c). The largest group comprising all but four plant ash beads from Komani and Lezha (*n* = 24), has very high magnesium contents (MgO > 3.5%), relatively low phosphorus levels and a much lower potassium to magnesium ratio compared to the four beads from Komani that clearly form a separate group (Table 2, Fig. 7). These characteristics are typical of plant ash glasses from Sasanian and Abbasid Mesopotamia (Mirti et al. 2009; Pion and Gratuze 2016; Henderson et al. 2016), an attribution that is confirmed by their low lanthanum and elevated chromium contents (Shortland et al. 2007). The four separate beads have on average higher titanium to zirconium ratios, clearly pointing to differences in the silica source and by extension different origins (Fig. 7a, b). Intriguingly, these four small beads (K_008, K_009, K_010, K_011) have been manufactured by cold-cutting in imitation of the Indo-Pacific ‘lada’ technique. Hence, they form typologically and compositionally a very tight group, suggesting a common provenance. No compositional parallels among published data of Near Eastern or Mesopotamian plant-ash glasses have been identified, neither for the silica source (higher alumina contents) nor for the alkali sources (lower calcium and higher potassium oxides levels) (Henderson et al. 2004, 2016; Henderson and Allan 1990; Gratuze and Barrandon 1990; Kato et al. 2010). A possible exception are some samples from Raqqa group 4 that are high in alumina and magnesia (Henderson 2003a). It has been argued that these might be the result of experimentation (Henderson et al. 2004).

**Fig. 8:**
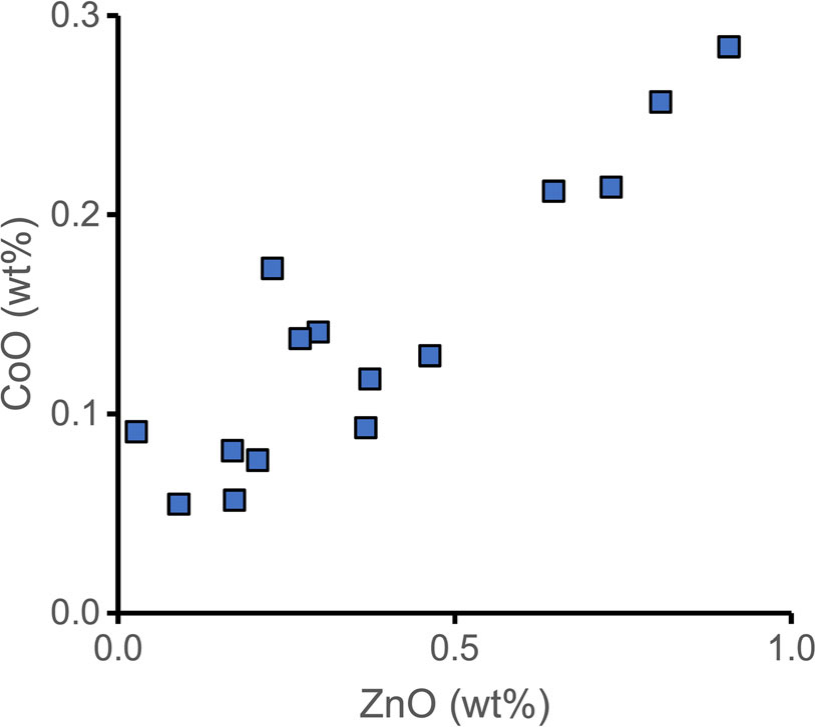
Correlation between cobalt and zinc in the blue beads of likely Mesopotamian origin

Some of the colourants represented among the plant-ash glass beads similarly confirm the chronological and geographical attribution of the groups. The cobalt in the blue beads is strongly correlated with zinc (Fig. 8). This type of zinc-rich cobalt is generally associated with Islamic glass making (Gratuze et al. 1992; Henderson 2003b; Wood 2012) and has been identified among the early Islamic glass from Tyre (Freestone 2002) and the al-Barsa beads (Robertshaw et al. 2010). The yellow and greenish yellow samples contain lead stannate pigments that occasionally show an increase in arsenic and antimony (Table 2). This type of lead stannate was previously identified in a set of Merovingian beads and in some tesserae from Durres (Neri et al. 2017).

The metal sandwich beads are in fact silver leaf glasses (Greiff and Nallbani 2008). They appear golden due to the brownish colour of the glass as a result of elevated manganese concentrations. The violet beads are coloured by manganese associated with iron, whereas the greens are coloured by copper and iron. The iron here is not correlated with titanium, suggesting its voluntary addition as colourant similar to a later Venetian recipe called ‘croco di ferro’, a combination of oxidised copper and reduced iron (probably iron sulphur) (Moretti 2012).

### Liturgical vessels

The four lamp fragments (Fig. 4) are likewise soda ash glasses, representing yet another silica as well as alkali source. They have lower magnesium, potassium and phosphorus concentrations than the small group composed of four beads but seem otherwise related. For example, even though they have higher absolute levels of both titanium and zirconium, the ratio between the two elements is similar. They show a similar composition to the glass artefacts from the monastery of Wadi al-Tur on the Sinai Peninsula dated to the ninth or tenth century (Kato et al. 2010).

The spindle-shaped *unguentarium* (K_025) is produced from soda ash glass with low alumina and titanium and higher calcium and zirconium contents. It is coloured with a cobalt colourant associated with indium, zinc and lead, commonly dated to the thirteen century (Gratuze et al. 1992). It has furthermore white filaments opacified with tin oxide, probably added as lead tin calx, a recipe that has been used likewise since the thirteenth century (Mason and Tite 2007).

## Discussion

The match between the typological and chemical analyses shows two different traditions, reflecting changes in the commercial networks sometime between the seventh and ninth or tenth century CE (Table 3). The wound beads are produced with natron glass opacified with tin oxide, coloured with a Roman cobalt ore or using lead, iron and manganese to obtain black glass, all suggesting a western production. The drawn beads instead show a Mesopotamian or Levantine soda ash glass signature, opacified with tin oxide, but coloured using cobalt positively correlated with zinc typical of Islamic glasses.

**Table 3:** Comparative analysis of typology and composition of beads

		seventh-century beads			ninth- to tenth-century			
Archaeological context		Lezha, T50, T146, T157			Komani, T319, T321, T340, T375, US1011, US1012, Lezha T34			
Technology		Wound bead	Black wound beads		Wound beads	Drawn segmented	Drawn, cold-cut (“lada” method)	Colourless Wound beads
			Ovoidal	Trilobed				
Chemical signature	Base glass	Natron glass (Foy 2, Roman Sb, cullet)	Recycled natron glass (Foy 2)		Mesopotamian soda ash glass		Levant soda ash	Recycled natron glass+ Egypt II
	Colourants Opacifiers	White: Cassiterite (SnO) Yellow: Lead stannate (PbSn) Blue: Cobalt-nickel Green: Copper	Lead iron (PbO, Fe_2_O_3_)	Lead iron Manganese (MnO)	White: Cassiterite (SnO) Yellow: Lead stannate Lead stannate with arsenic Blue: Cobalt-zinc		Green: Copper-iron Purple: manganese	
Provenance		Western or local production	Local production?	Local production?	Mesopotamian area		Levantine area	Local production or re-use

### Western beads in the seventh century

As mentioned earlier, the wound beads from Lezha have parallels among sixth- and seventh-century Merovingian and Lombard assemblages. They evidently reflect manufacturing techniques widely practices in western Europe. The lack of drawn segmented beads at Lezha is of particular importance as this appears to provide evidence that Lezha was cut off from the common trade routes through which eastern Mediterranean and Indo-Pacific beads reached western and northern Europe during this period. Similarities with the assemblages from some Danubian necropoleis (Egorkov 2006) and the Ionian coast of Calabria (Corrado and Verità 2012) indicate that local commercial networks were by contrast still flourishing. This may have been a direct result of the political situation in the seventh century, when the Byzantines had lost control of the Balkans, thus restricting the circulation of products due to the rupture of the cultural and political link between Illyricum and the Byzantine East.

The Lezha beads may have been either imported from further west or made regionally. The latter hypothesis is supported by the presence of the black beads high in iron and lead. Their chemical make-up is notably different from the Merovingian examples that have typically lower lead contents (9–12%) (Poulain et al. 2013; Mathis et al. 2013; Pion and Gratuze 2016). A possible exception are the beads from Maastricht that have on average similar lead concentrations (27%), but lower iron contents (about 1%) (Sablerolles et al. 1997). The black beads from Viminacium and Singidunum also have lead levels comparable to those from Lezha, but with a low iron content (Egorkov 2006). The existence of a different composition for each geographical area implies different, possibly local productions following a common manufacturing technique that made use of iron and lead oxides or metallurgical scrap. The iron and lead contents of the Lezha beads are close to the so-called Slavic potash rich high lead glasses (Steppuhn 1997; Bezborodov 1975), whereas they are clearly distinct from other medieval central European lead glasses (Mecking 2013) or from Islamic soda ash lead glass found in medieval Spain (Duckworth et al. 2015; de Juan Ares and Schibille 2017).

### Re-use of ancient beads

Three colourless wound beads from Komani found in ninth- to tenth-century graves were produced probably from cullet of older natron-type glass. The spherical wound bead with oculi (K_026) represents an exception in that it was made from a combination of sixth- to seventh-century Foy-2 type glass, decorated with a white glass of Egypt II characteristics, usually dated to the eighth or ninth century. Morphologically and compositionally similar beads were included in necklaces retrieved from Slovenian graves, where they typically assume a prominent position (Šmit et al. 2012). It is tempting to speculate that these re-used glass beads represent an heirloom passed down through generations. Respect for ancestors and its open display was an intrinsic trait of the conservative culture and local customs of the Balkans (Nallbani 2002). In fact, symbolic objects that once belonged to earlier generations are often found in the context of dressed inhumations, because burials were supposed to exhibit the identity of the deceased (Bougard et al. 2005). As specified in some Lombard wills, parts of necklaces and belts were offered to one’s heirs and mounted in new objects (La Rocca 2005).

### Long-distance trade in the ninth-tenth century

All the segmented drawn beads, most of the wound beads from Komani and the two medieval ones from Lezha have an Islamic chemical signature typical of glass from Mesopotamia (Henderson 2013). Despite the relative scarcity of archaeological evidence of production sites, the lack of any archaeological record of the production of segmented beads in western Europe (Arveiller-Dulong and Nenna 2011), suggests that these beads were imported from Mesopotamia under Abbasid rule. An Islamic, yet different provenance can be assumed for the cold-cut drawn beads produced with the ‘lada method’. These beads have higher alumina, soda and lanthanum contents than the Mesopotamian beads, but less than Indo-Pacific ‘lada beads’ (Pion and Gratuze 2016). This combination of a Near Eastern soda-rich plant ash signature with the ‘lada technique’ has not been previously identified, and possibly reflects the transfer of secondary working techniques from India to the Abbasid caliphate.

The presence of Islamic beads in south-western Illyricum during the ninth century testifies to a renewed commercial and cultural connectivity of the Balkans after the region had again come under Byzantine dominion (Cheynet 2006; Fine 1983). Similar observations had been made with respect to the ceramic finds from Butrint (Vroom 2008; Hodges et al. 2009; McCormick 2007). What is more, the exclusiveness of Islamic beads among the ninth- and tenth-century finds reinforces the idea of an Islamic monopoly (Callmer 1977). Textual sources, for example, recount how Arab merchants sold beads for one *dirham* apiece to the Vikings on the Volga in 922 CE (Simonsen 1981; Ahmad ibn Fadlan, *Risala*, 80–92). The evidence from Lezha and Komani confirm the wider currency of Islamic beads and the strategic position of the Balkans might in fact have been an important vector for this trade.

### Lamps and unguentarium

The chemical characteristics of the four vessel fragments reflect yet another source of supply. Compositionally, they resemble some Byzantine artefacts from a Coptic monastery on the Sinai Peninsula (Kato et al. 2010). Typologically the closest parallels for the snake trailed and large beaker lamps are found among middle Byzantine vessels, particularly from Thessaloniki, where secondary production workshops for this type of vessels have been identified (Antonaras 2008, 2010b).

The spindle-shaped unguentarium from Komani can be attributed to a post-thirteenth-century date, due to the very distinct cobalt source and opacification used. Cobalt associated with zinc and indium derives most certainly from the mines in Freiberg that were exploited above all between 1168 and 1250 (Seccaroni and Haldi 2016), while the lead tin calx technique was known since the thirteenth century (Mason and Tite 2007). This vessel is an imitation of an Abbasid typology that was particularly popular in the later Byzantine period (Antonaras 2008). The unusually high zirconium content excludes a Venetian provenance, meaning the unguentarium was either locally produced or imported from the eastern Mediterranean.

## Conclusion

The glass beads recovered from Lezha and Komani share characteristics of roughly contemporary bead assemblages from Europe and the Middle East. Specifically, the seventh-century samples from Lezha reflect compositional and typological trends comparable to western Europe, while the beads from Komani, dating to the ninth and tenth century, are clearly associated with Islamic bead making and a Mesopotamian supply. The earlier assemblage from Lezha is at the same time readily distinguished from those of other European sites in that no long-distance imports are evident. This chronological development mirrors wider economic and political changes in the region. During the Slavic occupation in the seventh and eighth century, Illyricum appears to be relatively isolated and cut off from the main trade routes connecting the eastern Mediterranean with western Europe. This is all the more astonishing, since the Balkans occupy a central strategic position along the traditional sea routes. The situation changes with the re-conquest of the Illyricum by the Byzantines in the ninth century, when long-distance trade with the Islamic east is revived. It also shows that Byzantium acted as a mediator for the exchange of beads, making the main trade axes in the Blakans safer. The Islamic beads arriving in Komani probably served a representative purpose analogous to Slavic customs more generally. These beads were after all grave goods, reflective of the cultural identity and status of the deceased. Islamic beads were evidently sought-after products and have dominated the Mediterranean and European market from Scandinavia to Morocco during the ninth and tenth century. This is in clear contrast to the ecclesiastical lighting devices recovered from Komani that are proposed to have been sourced from a Byzantine context instead.

## Electronic supplementary material

Table S1 LA-ICP-MS data of glass standards in comparison with published values. Corning A values correspond to Vicenzi et al. 2002; NIST 612 correspond to Jochum et al. 2011. Oxides, including chlorine are given in weight %, elements are given as ppm. a After Wagner et al. 2012; b After Hollocher et al. 1995. (XLSX 11 kb)
